# A 0.3 V PNN Based 10T SRAM with Pulse Control Based Read-Assist and Write Data-Aware Schemes for Low Power Applications

**DOI:** 10.3390/s21196591

**Published:** 2021-10-02

**Authors:** Ming-Hwa Sheu, Chang-Ming Tsai, Ming-Yan Tsai, Shih-Chang Hsia, S. M. Salahuddin Morsalin, Jin-Fa Lin

**Affiliations:** 1 Department of Electronic Engineering, National Yunlin University of Science and Technology, Douliu City 64002, Taiwan; sheumh@yuntech.edu.tw (M.-H.S.); m10813014@gemail.yuntech.edu.tw (C.-M.T.); M10513211@gemail.yuntech.edu.tw (M.-Y.T.); hsia@yuntech.edu.tw (S.-C.H.); s.morsalin10@gmail.com (S.M.S.M.); 2 Department of Information and Communication Engineering, Chaoyang University of Technology, Wufeng District, Taichung City 413310, Taiwan

**Keywords:** low power, bit-cell, static random-access memory, sub-threshold voltage, half-select disturbance

## Abstract

An innovative and stable PNN based 10-transistor (10T) static random-access memory (SRAM) architecture has been designed for low-power bit-cell operation and sub-threshold voltage applications. The proposed design belongs to the following features: (a) pulse control based read-assist circuit offers a dynamic read decoupling approach for eliminating the read interference; (b) we have utilized the write data-aware techniques to cut off the pull-down path; and (c) additional write current has enhanced the write capability during the operation. The proposed design not only solves the half-selected problems and increases the read static noise margin (RSNM) but also provides low leakage power performance. The designed architecture of 1-Kb SRAM macros (32 rows × 32 columns) has been implemented based on the TSMC-40 nm GP CMOS process technology. At 300 mV supply voltage and 10 MHz operating frequency, the read and write power consumption is 4.15 μW and 3.82 μW, while the average energy consumption is only 0.39 pJ.

## 1. Introduction

Mostly, the SRAM occupies a large amount of layout area in the system on chip (SoC) that affects the system’s integrity [1,2]. The SoC may classify into two categories: high-speed and low-power consumption. Low voltage operation and long-term service are becoming more challenging for IoT and portable applications [3,4]. Therefore, developed a stable [5] SRAM architecture for low voltage and sub-threshold operation. The difficulties are manufacturing variation and impair circuit’s performance while decreasing the voltage. Traditional 6T SRAM [6] is the most basic configuration and proposed local bit-line 6T [7] achieved low power operation, but there have limitations for half-selected operation and read error. A read-decoupled (RD) 8T SRAM was proposed [8] to avoid the read error. This architecture has much better read static noise margin (RSNM) performance during the single-ended read operation mode. Furthermore, the RD8T design shows the half selected causing pseudo read while write operation.

Many SRAM bit-cell design [9,10,11,12,13,14,15,16,17,18,19,20,21,22] have been presented to enhance circuit stability for robust low voltage/power operation. In [9,10,11,12,13,14,15], are provided stacked transistors to solve the problem of half-selected read error, but they bring worse write capacity. In order to overcome this problem, a word line boost circuit technology to improve the write capability are presented in [16,17]. However, the problem of data contention (cross-coupled inverter structure) still exists. Therefore, these designs need additional write assist techniques to improve write margin [18,19]. Another solution is to cut off the cross-coupled inverter’s feedback path to improve the write capability, but the half-selected write stability still affected [12,13]. In [21], uses a single-ended write structure to reduce circuit complexity, but each column needs to use an extra read feedback circuit to enable it to operate stably in the subthreshold region, but the number of transistors will still be as high as 12.

In summary, the traditional 10T (or higher transistor count design) bit-cell provides better circuit stability, but usually at the cost of their write-ability and limit their work VDD_min_. Therefore, write auxiliary circuits are usually required, such as a VDD/GND boost circuit scheme, which leads to the need for additional power overhead. Hence, for robust subthreshold operation, we proposed a SRAM bit-cell design and employed pulse control read-assist circuit, as well as write data-aware schemes to cut off the pull-down channel for improving the write ability and eliminating read error without any boost circuit when keeping transistor-count in 10. The main contributions of this work are summarized as follows:

❖ The proposed 10T SRAM design solves the half-selected problems;
❖ The read decoupling technique increase the read static noise margin;
❖ The write data-aware techniques cut off the pull-down path and achieved low leakage power.

## 2. Proposed 10T SRAM Architecture

### 2.1. Memory Cell Design with 10T

**Figure 1:**
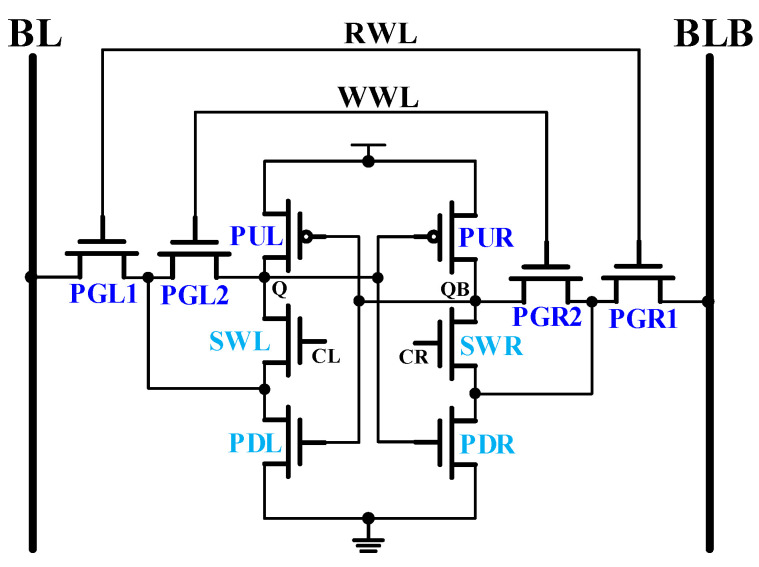
The proposed 10T SRAM bit-cell design.

Figure 1 shows the proposed 10T SRAM bit-cell design. The cross-coupled latch consists of two PMOSs (PUL, PUR) and four NMOSs (SWL, PDL, SWR, PDR). Therefore, the proposed design has been named as PNN-10T SRAM cell. In this design, both CL and CR signals pulse control read-assist and write data-aware schemes control SWL and SWR NMOSs to improve the read and write speed performance. The write word line (WWL) also controls both PGL2 and PGR2 to write the input data. Additionally, the read word line (RWL) is used to control PGL1 and PGR1 to read the data from latch or write the data from bit lines (BL, BLB).

**Figure 2:**
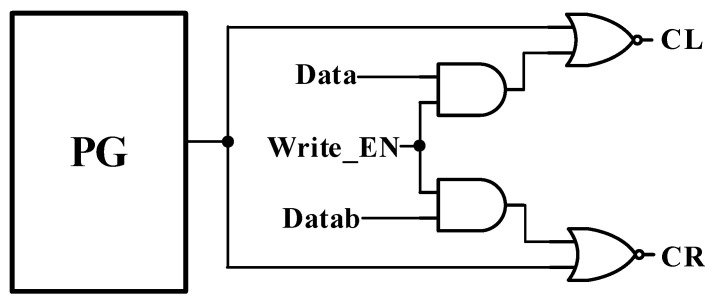
Pulse control read-assist and write data-aware schemes.

Figure 2 shows the control signals CL and CR pulse control read-assist and write data-aware schemes operation. When write EN is set to ‘0’, the CL and CR control signals are set to ‘1’, allowing the cell to keep the data in hold mode. The write EN remains ‘0’ during the read operation, while the pulse generator (PG) controls CL and CR, with the pulse being cut off by a pull-down (SWL/SWR) transistor. This can isolate Q/QB from bit lines BL/BLB for a short time. Finally, the write_EN variable is set to ‘1’, so that the CL and CR values are determined by external data (Data and Datab) in the write operation.

Table 1 shows the truth table of the proposed 10T SRAM bit-cell for standby state signal settings while read, write operation mode, and the control signal circuit is used to complete the switching of each signal in the cell under different situations. 

### 2.2. Write Mode Operatio

**Figure 3:**
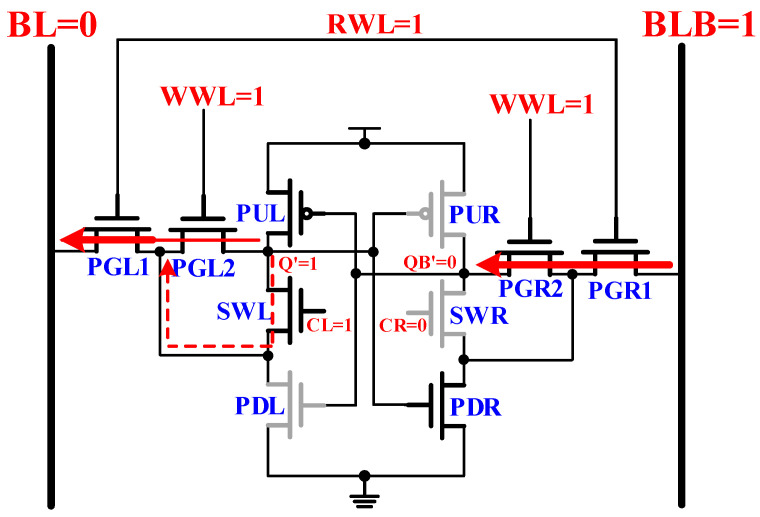
The write operation of proposed 10T SRAM cell.

The write mode operation of the proposed 10T SRAM cell is shown in Figure 3. The RWL and WWL set the value of ‘1’, whereas the BL and BLB write data. The write data-aware methods shut off the pull-down route (CL/CR) on one side of the latch for data sensing. To write data ‘0’, for example, the discharge route node Q will pass via PGL2 and PGL1 on its way to BL. At the same time, the left control signal (CL) will set ‘1’ so that SWL can continue to start and provide additional write current from SWL to PGL1. The cell flips quickly, and the right control signal (CR) turns off the SWR, thus cutting off the latch pull-down path. 

### 2.3. Read Mode Operation

**Figure 4:**
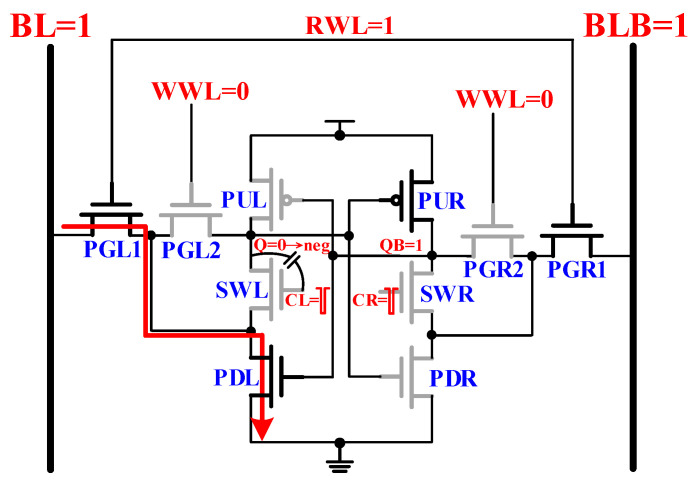
The read operation of proposed 10T cell.

**Figure 5:**
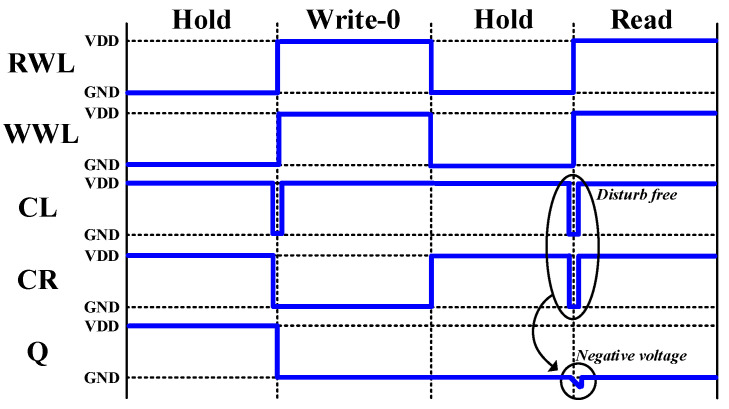
Time-domain waveform diagram of proposed 10T cell.

The cross-coupled latch discharges the proposed 10T SRAM cell by the following arrows direction, as shown in Figure 4. The read path links PGL1/PGR1 and PDL/PDR to ground. During the read operation, the pulse control read-assist technology provides a dynamic read decoupling method with PG circuit short pulse signal (CL/CR). If the stored data Q = ‘0’ and QB = ‘1’, then the path is pull-up to the cross-coupled latch because the PUR is active, and the CL/CR will be turn off for a short time. The output negative voltage connects the parasitic capacitance Cgs, and SWL, SWR is off, thus helping the proposed design is unaffected by the pull-down path. Figure 5 depicts the post-layout simulation waveforms of the proposed design.

**Figure 6:**
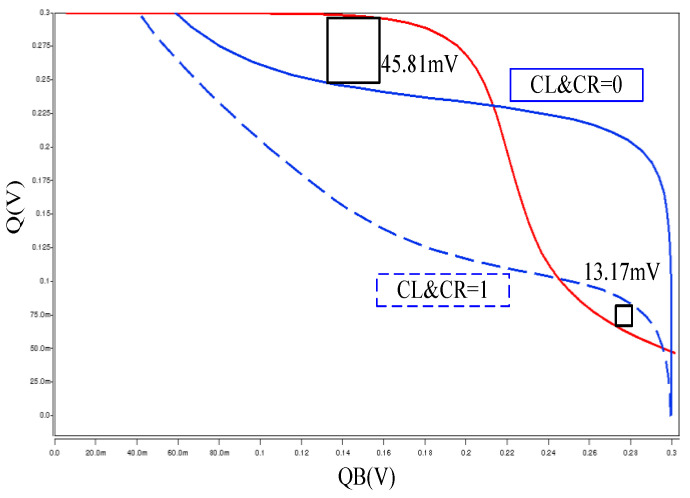
RSNM of proposed 10T cell.

Figure 6 displayed the RSNM simulation result, and the authors carried out two cases results of RSNM at 0.3 V operating voltage to further validate their findings. The red line represents the overlapping of the two cases. The first case does not turn off SWL and SWR (CR and CL = ‘1’). Considering the operation, node QB becomes affected, and its RSNM is only 13.17 mV (represented by blue dotted line), which means it is easy to flip during the sub-threshold voltage operation. Additionally, the second case is when the read operation is in progress, SWL and SWR (CR and CL = ‘0’) temporarily turn off, node QB is enabled to be isolated from BLB and reduces error. The RSNM rises to 45.81 mV, this dynamic reading and decoupling method is a very important circuit scheme for the proposed structure.

### 2.4. Write Half-Selected

**Figure 7:**
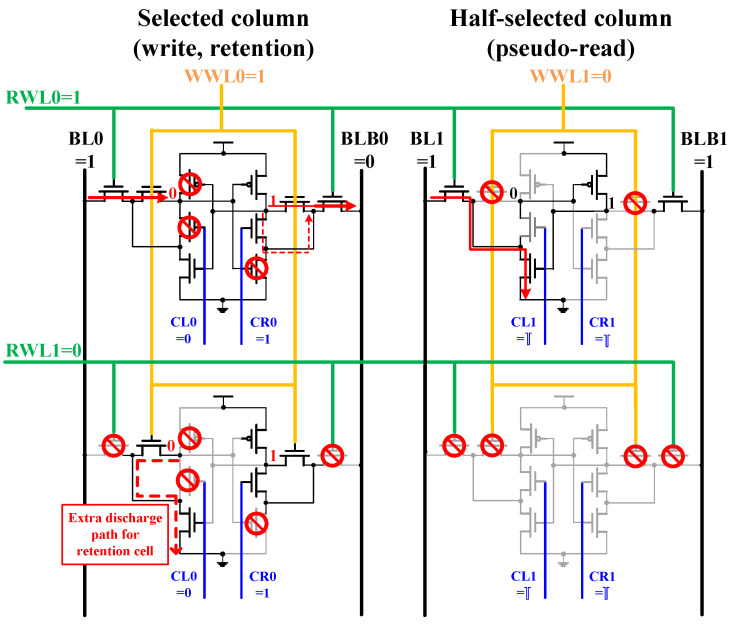
Write half-selected analysis of the proposed design.

Figure 7 depicts the row and column half-selected cell writes operation for the proposed design. The signal RWL0 becomes active in the write operation, and the short pulse removes the erroneous read error by performing a pseudo-read of the column half-selected cell. The short pulse consumes very little power through the Cgs of the transistor. In addition, negative voltage compensation is also produced, which improves the stability of cells. However, RWL1 is inactive in the row half-selected cell. The storage nodes Q and QB become separated that lowering BL and BLB errors. The write data-aware methods cut off the pull-down path while executing a write operation, which affects the stability of the row and half-selected cell. In addition, the stored data are the same, which cut off the drop-down path that keeps the state cell storing ‘0’. Fortunately, the WWL0 initiates the drop-down route (red dotted line), which retains the state cell in the half-selected state. The proposed design has the better stability whether it is a half-selected cell in a row or a column mode.

**Figure 8:**
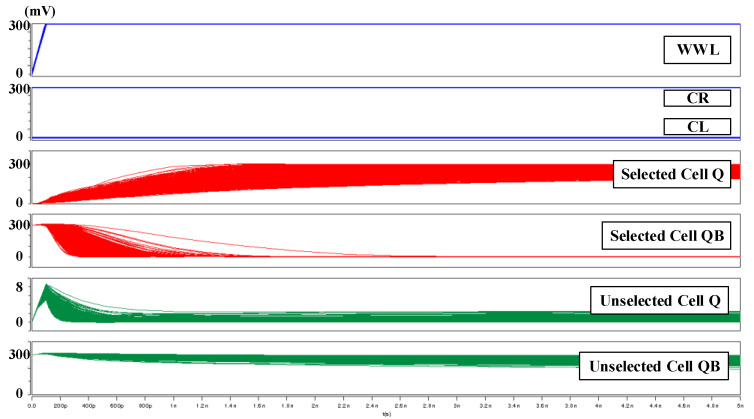
The 3000 times Monte Carlo simulation waveforms for column half-selected condition.

Figure 8 shows 3000 times Monte Carlo simulation waveforms for the column half- selected cell at 0.3 V supply voltage. When WWL is active, the selected cell continues to flip successfully. The node Q of the selected cell is pushed down to 0 V, providing a strong discharge path for the unselected cell.

## 3. Layout Design and Simulation Results

**Figure 9:**
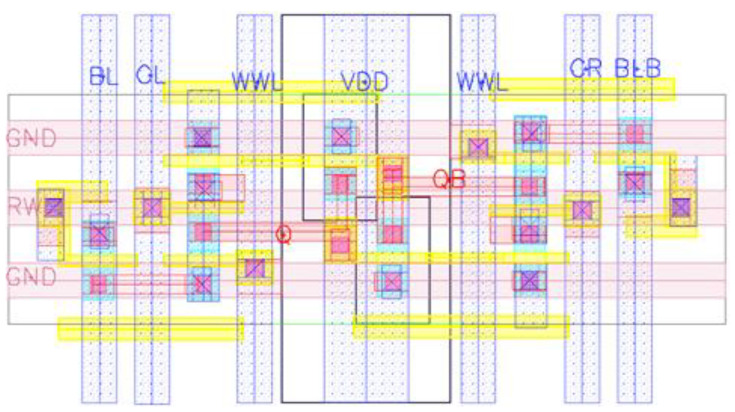
Layout schematic of the proposed 10T design.

Figure 9 shows the layout design of the proposed 10T SRAM architecture implemented on TSMC 40 nmGP using the CMOS process technology. The layout layer has been used from diffusion to metal 3, and the size of the layout area is 3.28 μm^2^ (1.2 μm × 2.73 μm).

**Figure 10:**
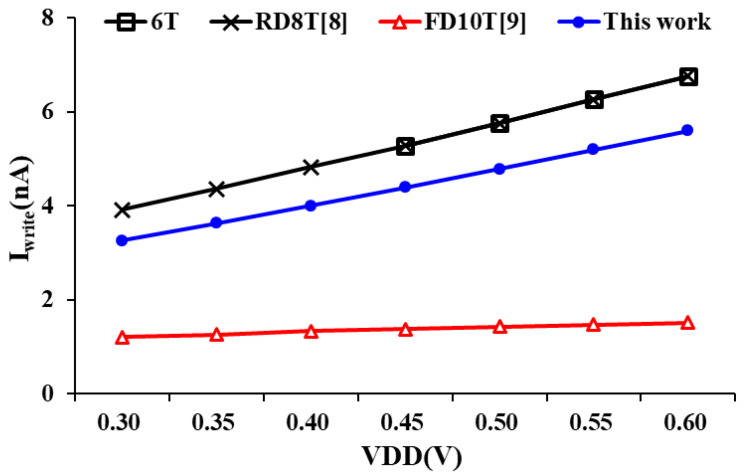
Bit-cell write current comparison at different voltages.

Figure 10 compares different bit-cells write ‘0’ current at different voltages. For example, the 6T and RD8T [8] do not have stack pass gate transistors, so both maintain the same write current throughout the write operation. However, both are affected by the half-selected error and unable to operate at low voltages. Alternatively, the write data-aware techniques have been included in the proposed design to block the pull-down path for write ‘1’ in advance, and the switch becomes active for write ‘0’ at the same time. This technique adds a path from SWL, PGL1 to BL. The write data-aware schemes make better and stable write ability for the proposed design.

**Figure 11:**
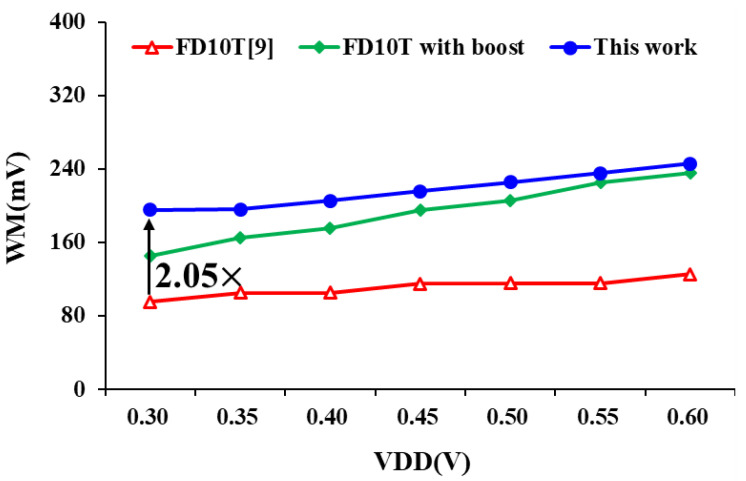
Write margin of bit-cell at different voltages.

Figure 11 shows the comparison of write margins (WM) under different supply voltages. FD10T design [9] records the results of with or without VDD boost circuit scheme. Because the proposed design adopts the write data-aware technique, it not only provides an additional current path when writing 0, but also successfully cuts off the pull-down path (writing 1). Post-layout simulation results show that, compared with FD10T [9], the proposed design can increase the write capability by more than 2 times without any boost circuit assistance.

**Figure 12:**
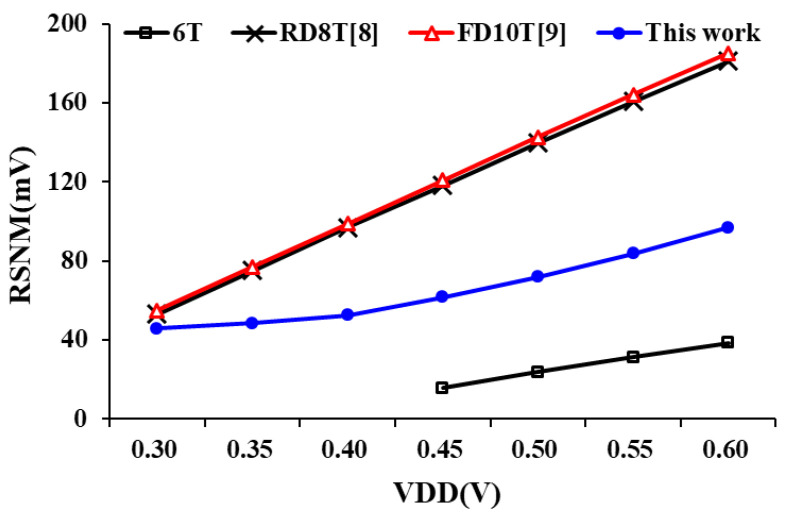
RSNM of bit-cell operates at different voltages.

The RSNM of the proposed design read decoupling technique, which isolates the Q/QB node from pulses for a short period, is shown in Figure 12. By discharging the stacked NMOS, the read path substantially enhances the RSNM. The RSNM curve of the proposed design delivers low voltage and small attenuation. When the working voltage is increased, the swing of all nodes increases as well, which reflects noise tolerance.

**Figure 13:**
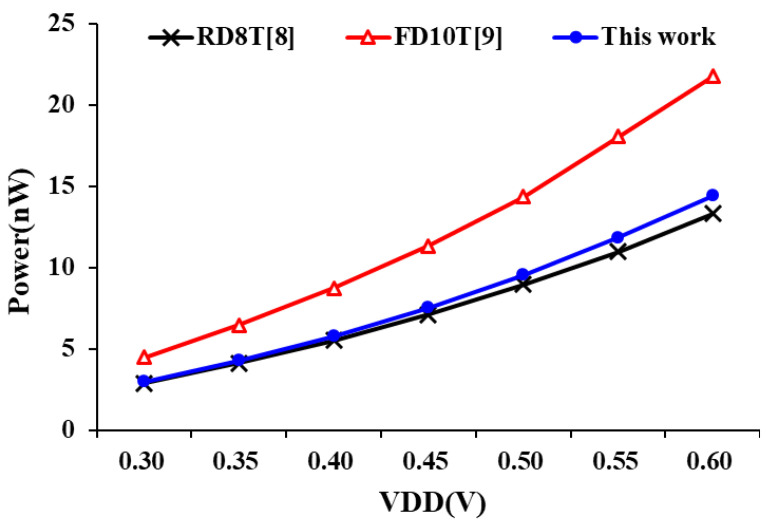
Power consumption of bit-cell at different supply voltages.

**Figure 14:**
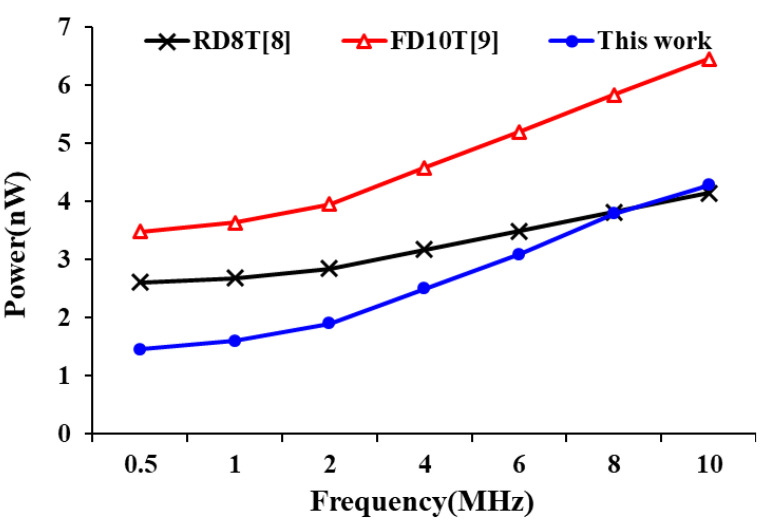
Power consumption of bit-cell at different operation frequency.

In the power consumption performance, we recorded the power consumption of these designs at different supply voltages and different operating frequencies. Figure 13 shows the power consumption of the each SRAM bit-cell design at supply voltages range from 0.3 to 0.6 V. As we can see, the proposed SRAM 10T bit-cell has similar power consumption to RD8T [8] design and saves over 30% compared to FD10T [9]. Figure 14 also shows the power performance of these designs at different frequencies (operation voltage is set at 0.3 V). The proposed design is more advantageous at low frequencies (in this case, the leakage power is the dominant factor) and can greatly reduce power consumption when compared with other designs. When the operation frequency is 10 MHz, the power consumption of the proposed design is only 4.29 nW.

**Figure 15:**
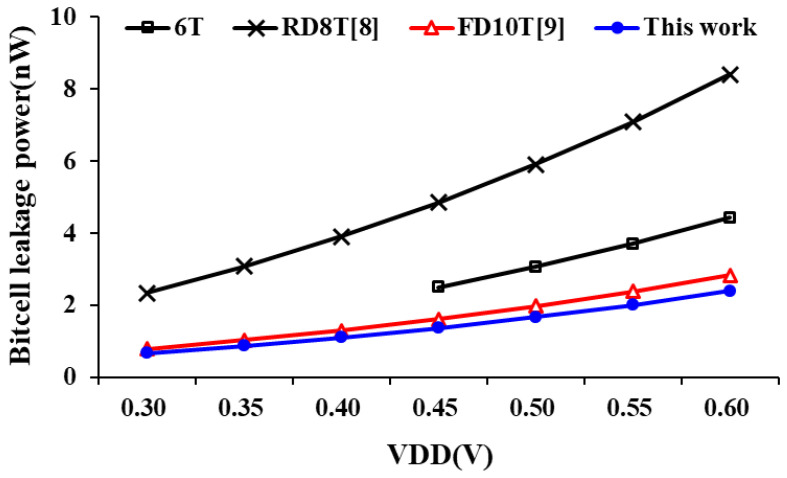
Comparison of bit-cell leakage power consumption.

Finally, Figure 15 shows these SRAM bit-cell leakage power consumption comparison at different supply voltages. Due to the stacked PG circuit, the proposed design has overlying pull-down paths in the cross-coupled latch to reduce leakage current. As a results, the proposed design has the lowest leakage current power consumption.

## 4. Chip Implementation

**Figure 16:**
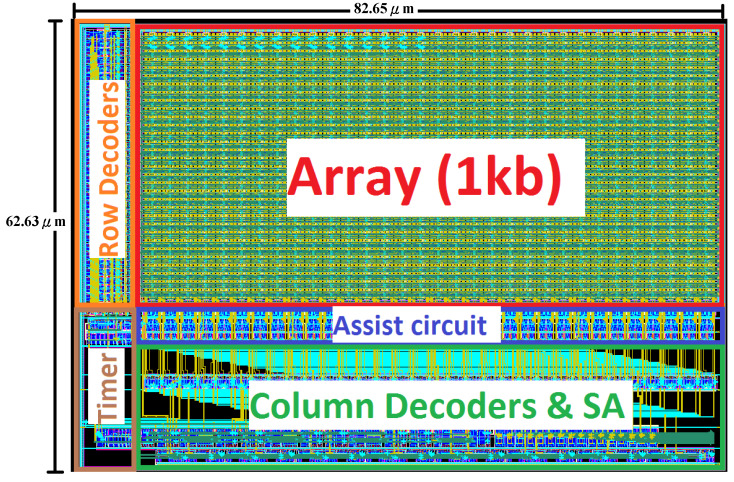
The 1 kb-array layout of the proposed design.

Figure 16 shows the implemented 1 kb SRAM macros (32 rows × 32 columns) layouts. The layout area is 5401.84 μm^2^ (86.25 μm × 62.63 μm). The composition of this architecture is cut into 4 banks, each bank is 256 bits (32 rows * 8 columns), every time read and write operation is required, one of the banks will be selected, and the 8 bits write or 8 bits readout.

**Figure 17:**
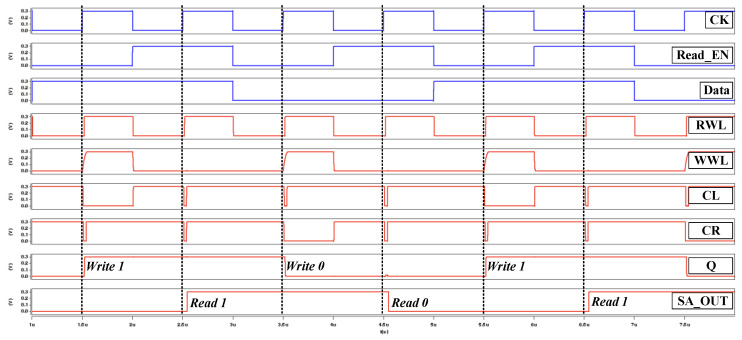
Post-layout simulation waveforms @300mV/TT corner.

The post-layout simulation waveforms of test chip is shown in Figure 17 at 300 mV operation voltage. This test parameter confirms that by using the pulse control read-assist and write data-aware schemes our design can successfully reduce the read disturbance to operate at the sub-threshold operation.

Table 2 lists key features of several subthreshold SRAM designs for comparison. The traditional 6T SRAM design is also included. Due to the above-mentioned circuit problems, without the assistance of other auxiliary circuits, his operating voltage cannot be lower than 0.6 V. Since the proposed design 10T SRAM bit-cell design does not require any boost circuit assistance and uses stacked transistors circuit structure, our design not only has a better chip area (12-transistor vs. 10-transistor) but also reduces energy consumption by 79.58% when compared with PCA12T design [10]. Thus, our 10T SRAM bit-cell is the most efficient design for low power/voltage application, i.e., mobile devices and bio implants.

## 5. Conclusions

This paper presents a novel 10T bit-cell design for robust low voltage and power operation. The proposed design uses a bit-interleaving SRAM array, allowing the column half-selected cell to acquire the discharge channel from floating during the write operation. The proposed design has robust performances in read, write, and hold operation at sub-threshold voltage. The simulation results of 40 nm 1-kb SRAM at 0.3 V/10 MHz demonstrate that the power consumption for read and write operation is just 4.15 μW and 3.82 μW, respectively. Due to a stacked pull-down circuit scheme has also been used to reduce leakage current. The retention power of 3.64 μW can be achieved at 0.3 V.

## Figures and Tables

**Table 1:** Proposed 10T SRAM cell truth table performance.

	Hold	Read	Write-0	Write-1
Write_EN	0	0	1	1
BL/BLB	1/1	1/1 (floating)	0/1	1/0
RWL	0	1	1	1
WWL	0	0	1	1
CL/CR	1/1	Pulse	1/0	0/1

**Table 2:** The SRAM characteristics comparison table.

Characteristics	6T [22]	FD10T [9]	DFL10T [11]	PCA12T [10]	Proposed10T
Process	28 nm	90 nm	28 nm	40 nmGP	40 nmGP
Assist Scheme	Optimized Peripheral	WordLine boost	No necessary	DAPC	PCR + WDA
VDD_MIN_	0.6 V	160 mV	250 mV	350 mV	300 mV
Capacity	128-kb	32-kb	32-kb	4-kb	1-kb
Frequency @VDD_MIN_	20 MHz	500 Hz	30 kHz	11.5 MHz	10 MHz
Read Power (μW)	2800	0.123	0.088	22.0	4.15
Write Power (μW)			0.087		3.82
Energy/Access (pJ)	140	246	2.92	1.91	0.39
Leakage Power (μW)	N/A	0.36 @ 6 °C	0.05 ^*1^	17.38	3.64

^*1^:The large negative bias VSG of power gating.

## Data Availability

The data presented in this study are available on request from the corresponding author.
